# Recast Layer-Induced Fatigue Degradation in High-Speed EDM Microholes: Experimental Characterization

**DOI:** 10.3390/ma18091985

**Published:** 2025-04-27

**Authors:** Yaou Zhang, Qian Zheng, Zeyu Wu, Hualin Liao, Yifan Lu, Juncheng Lu

**Affiliations:** State Key Laboratory of Mechanical System and Vibration, Shanghai Jiao Tong University, Shanghai 200240, Chinaliaohualin@sjtu.edu.cn (H.L.); sh_luyifan@sjtu.edu.cn (Y.L.); junchenglu@sjtu.edu.cn (J.L.)

**Keywords:** high-speed electrical discharge machining (EDM), small holes, recast layer, fatigue damage, fatigue life

## Abstract

High-speed electrical discharge machining (EDM) is crucial for drilling aerospace components, but the fatigue effects of its recast layer are still not well understood. This study investigates the fatigue behavior of high-speed EDM-processed specimens using ultrasonic fatigue testing and microscopic analysis. The recast layer showed a 20.4% increase in hardness and a 16.5% decrease in elastic modulus compared to the base material. Fatigue cracks originated from microcracks, pores, and inclusions within the recast layer, as well as at its interface with the substrate. Microscopic crack initiation was influenced by defect interactions, while macroscopic crack initiation occurred near the maximum hole diameter perpendicular to the loading direction due to stress concentration. The specimens exhibited bimodal fatigue life: shorter lifetimes were observed when macroscopic stress concentrations overlapped with recast layer defects such as cracks and voids, while defect-free regions significantly extended durability. The non-uniform distribution of the recast layer critically links microstructural heterogeneity to variations in fatigue failure. These findings highlight how recast layer characteristics influence crack nucleation and life variability in EDM-processed components, offering valuable insights for optimizing machining parameters to reduce fatigue risks in critical aerospace applications.

## 1. Introduction

GH3536 (Hastelloy X), a high-performance superalloy, is extensively used in critical components of aircraft engines, such as combustion chambers and turbine blades, due to its exceptional high-temperature mechanical properties and oxidation resistance [1]. However, during the operation of aircraft engines under extreme conditions, the surface temperature of these components can reach levels far beyond the material’s designed tolerance limits. Although surface coating technologies are commonly adopted to enhance the high-temperature resistance of components, their performance deteriorates significantly in environments where the temperature exceeds 2000 °C. To tackle this challenge, film cooling techniques have been introduced to insulate heat and reduce surface temperatures [2], thereby extending the service life of these components.

High-speed electrical discharge machining (EDM) is widely adopted for manufacturing small holes and other microstructures due to its efficiency and cost-effectiveness [3]. During the EDM process, the rapid heating and subsequent cooling of the workpiece material lead to the formation of a brittle recast layer (RL) on the surface [4]. This RL significantly modifies the microstructure of the hole walls and introduces a complex network of micro-defects, such as pores, cracks, and amorphous regions. These micro-defects act as stress concentrators, severely degrading the surface integrity and substantially reducing the fatigue performance of the machined components. Tai et al. [5] demonstrated that among the various defects generated on surfaces processed by EDM, surface cracks exert the most profound influence on the mechanical properties of the components. Zhang et al. [6] proposed that the reduction in fatigue strength is directly proportional to the thickness of the recast layer and the peak-to-valley roughness formed during the solidification of the molten material in the EDM process. Additionally, Zhang’s study established a positive correlation between the fatigue life and the surface hardness of the recast layer, indicating that a higher surface hardness of the RL could potentially improve the fatigue resistance to some extent. Macoretta et al. found that the fatigue life of small-hole structures fabricated by EDM is approximately 8% shorter compared to those processed by mechanical methods [7]. However, current research predominantly concentrates on the macroscopic aspects, such as the thickness of the RL and surface roughness, while the underlying mechanisms of fatigue crack initiation and distribution, particularly under high-cycle and very-high-cycle fatigue conditions, remain inadequately investigated. These mechanisms are crucial for optimizing the EDM process to improve the fatigue performance of components, especially in applications where high-reliability and long-term durability are required, such as in aerospace and automotive industries.

To address this, efficient experimental methods are crucial. Traditional hydraulic servo fatigue testing (frequency ≤ 100 Hz) is inadequate for evaluating aircraft engine components under very-high-cycle fatigue conditions. For instance, completing 10^9^ cycles at 50 Hz requires over 100 days [8]. Given the high-frequency vibrational loads experienced by turbine blades [9,10], more efficient testing methods are needed. Ultrasonic fatigue testing (UFT), operating at 20 kHz, reduces the time for 10^9^ cycles to 12 h [11], providing a powerful tool for studying RL failure mechanisms under high-cycle and very-high-cycle fatigue conditions. Combining UFT with microstructural analysis of EDM-processed RLs offers new insights into fatigue crack initiation.

It is still unclear whether the test results at a certain frequency are applicable to other materials at other frequencies [12]. This frequency effect difference shows significant characteristics in various metal materials. Researchers have revealed several key mechanisms by comparing traditional low-frequency (10–100 Hz) and ultrasonic high-frequency (20 kHz) fatigue tests: In the study of low-carbon steel systems, Guennec et al. [13] studied the effect of frequency on the crack initiation mechanism of JIS S15C low-carbon steel and found that the change in crack initiation mode is directly related to the longer fatigue life at ultrasonic frequency. Tsutsumi et al. [14] conducted ultrasonic fatigue tests at 20 kHz and conventional tension–compression fatigue tests at 10 Hz. The results showed that the crack growth rate in ultrasonic fatigue tests was lower than that in conventional tension–compression fatigue tests, mainly because the size of the plastic zone at the crack tip in ultrasonic fatigue tests was significantly smaller than that in tension–compression fatigue tests, which explains the reduction in crack growth rate. Nonaka et al. [15] found that the fatigue limit at 19.8 kHz was significantly higher than that at 10 Hz, which may be attributed to the rapid strain in ultrasonic fatigue tests increasing the yield strength of the material. In the field of high-temperature alloys, Zhang et al. [16] showed that the life of Inconel 718 specimens exhibited a positive frequency correlation when the loading frequency was increased from 52.5 Hz to 20 kHz. However, Morrissey et al. [17] found an anomaly in single-crystal nickel-based superalloys: the fatigue life data at 20 kHz decreased by about 20% compared to that at 60 Hz, but the failure mechanism remained highly consistent, suggesting that the intrinsic fatigue behavior did not change essentially. The “slip inhibition” theory proposed by Muhammad et al. [18] provides a reasonable explanation for such phenomena: high-frequency loading delays the damage process by reducing the amount of slip accumulation per unit time, thereby extending the overall life. From the above analysis, it can be seen that there are differences in the fatigue life analysis results under ultrasonic loading and traditional loading. The research on the influence mechanism of the material crystal structure has made important breakthroughs. The crystallographic model established by Youshi et al. [19] shows that body-centered cubic (bcc) lattice type materials (such as low-carbon steel) are sensitive to frequency effects, while hexagonal close-packed (hcp) lattice type materials and face-centered cubic (fcc) lattice type metals are insensitive to frequency effects. Yang et al. [20] analyzed that the fatigue life of aircraft turbines is about 10^10^ cycles, with low stress amplitude but high frequency, and the ultrasonic method can effectively evaluate the ultra-high cycle fatigue life of blades under non-synchronous vibration. For hcp structure nickel-based superalloy materials widely used in the aviation field, their frequency insensitivity gives ultrasonic testing unique advantages, so the ultrasonic fatigue method can be used for high-cycle and ultra-high-cycle life evaluation.

In this study, UFT was systematically employed to analyze the microstructural and physical properties of RLs in microholes fabricated via EDM. The primary objective was to precisely identify the crack initiation sites and comprehensively understand their distribution patterns across diverse defect types. By meticulously constructing S–N curves, the influence of RLs on crack initiation was quantitatively evaluated. For the first time, the contributions of pores, cracks, and amorphous regions within the RLs to fatigue crack initiation were systematically investigated. This research not only fills significant gaps in the understanding of fatigue crack initiation and distribution mechanisms but also offers crucial theoretical and technical guidance for optimizing EDM parameters.

## 2. Experimental Procedure

### 2.1. Workpiece Design

In this study, GH3536 superalloy sheets (Jinhe Special Material Manufacture Co., Ltd., Wenzhou, China), which had undergone a 2-h solid-solution treatment at 1175 °C, were chosen as the base material. The chemical composition of the alloy is presented in Table 1.

To isolate and study the independent influence of EDM recast layers on fatigue life, a simplified geometric specimen design was implemented. A through-hole with a diameter of φ 0.75 ± 0.02 mm was machined at the center of each sheet. During the machining process, the discharge energy was precisely controlled to ensure the formation of a typical recast layer structure on the hole walls.

**Figure 1:**
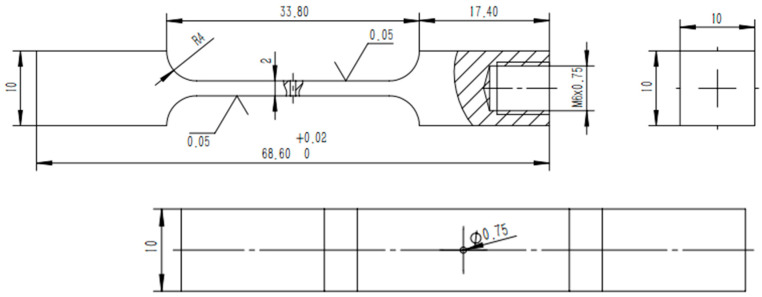
Specimens with film cooling holes.

This study utilizes an ultrasonic fatigue testing system to apply high-frequency alternating loads to metal specimens based on the principle of resonance. To ensure stable resonance at the target frequency of 20 kHz, the axial geometric dimensions of the specimens are systematically adjusted through parametric modeling. The dynamic characteristics of the specimens are iteratively optimized using Finite Element Modal Analysis (FEMA) based on the physical properties of the base material (as outlined in Table 2). Numerical simulations reveal that adjusting the specimen length to 68.6 mm results in a natural frequency of 20,172 Hz for the 13th mode, with a relative error of less than 1% compared to the preset excitation frequency. This meets the frequency domain matching requirements for ultrasonic fatigue testing [21]. This optimization step was crucial as it enabled the establishment of a standardized fatigue evaluation model, as depicted in Figure 1. By following this approach, this study could accurately assess the impact of the EDM-induced recast layers on the fatigue performance of the GH3536 superalloy specimens.

### 2.2. Workpiece Manufacturing

Standard specimens were initially machined using a machining center (VH-85, manufactured by Qiaofeng Intelligent Equipment Co., Ltd., Dongguan, China). This machining process was followed by grinding using a grinding machine (JGS-510AHD, produced by Zhongquan CNC Technology Co., Ltd., Shanghai, China). To eliminate visible scratches, the specimens were then polished with sandpaper ranging from 400 to 2500 grit.

To explore the impact of the recast layer on ultrasonic fatigue, micro-holes were fabricated on these pre-processed specimens. A six-axis high-speed EDM machine (Suzhou Zhonggu Industry Co., Ltd., Suzhou, China) was employed for this purpose. The key parameters of the high-speed EDM process, such as electrode diameter (D), pulse duration (Ton), pulse interval (Toff), peak current (IA), capacitance (C), and electrode rotation speed (r), were used to machine through-holes with a diameter of approximately 0.75 mm on fatigue specimens, as detailed in Table 3. To guarantee experimental consistency, all specimens were processed in a single batch under identical conditions. This approach ensured that any variations in the results could be primarily attributed to the effects of the recast layer rather than differences in specimen preparation or processing.

### 2.3. Experimental Procedure

**Figure 2:**
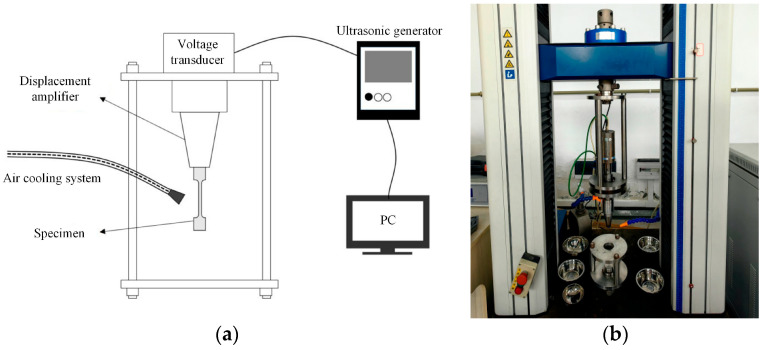
(**a**) Ultrasonic fatigue test equipment schematic diagram and (**b**) Ultrasonic fatigue test equipment.

All fatigue tests were carried out using an ultrasonic fatigue testing system independently developed by Sichuan University, as depicted in Figure 2. This system mainly comprises an ultrasonic generator, transducer, control system, and cooling system. Notably, it is equipped with a cold air device to maintain a stable temperature of 20 °C ± 5 °C throughout the testing process. The tests were conducted at room temperature, with a stress ratio (R) set to −1 and a loading frequency (f) of 20 kHz.

**Figure 3:**
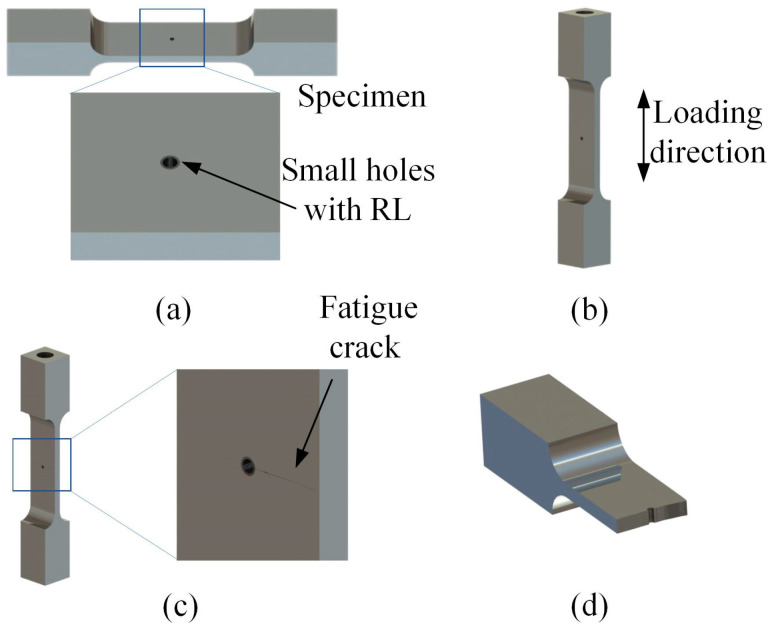
Flow of ultra-high cycle fatigue experiments: (**a**) small holes with a recast layer, (**b**) loading direction, (**c**) fatigue crack, (**d**) fracture analysis.

During the experiment, the specimen in Figure 3 was installed on the displacement amplifier of the fatigue testing machine in Figure 2 via a threaded connection. The maximum stress was applied by controlling the vibration amplitude of the ultrasonic waves of the fatigue testing machine. For specimens featuring recast layers, specific loading stresses were applied: 330 Mpa, 280 MPa, 230 MPa, 220 MPa, 215 MPa, 210 MPa, and 205 MPa. To reduce data variability, 3–5 specimens were tested at each stress level. The test procedure is graphically illustrated in Figure 3. During the tests, specimens demonstrated significant crack failure at the micro-hole location, with the cracks oriented perpendicular to the stress loading direction.

As the failed specimens no longer satisfied the ultrasonic resonance condition, cracks could not propagate to cause complete fracture by the end of the test. To examine the fatigue fracture surface after the crack has propagated, researchers cooled the specimen and then struck it. This additional test step ensured that the fracture characteristics could be comprehensively examined, contributing to a more in-depth understanding of the fatigue failure mechanisms related to the recast layer on the specimens.

### 2.4. Sample Observation Process

To observe the recast layer on the surface of high-speed EDM parts, a systematic sample preparation and analysis process was carried out. First, the samples were embedded. Subsequently, a machining center was used to remove half of the hole in each sample. This step was crucial as it exposed the cross-section of the hole where the recast layer was to be examined.

After the partial hole removal, the remaining portion of the sample was ground and polished using 400-grit sandpaper. This initial polishing step aimed to create a relatively smooth surface for better optical observation. The dimensions and distribution of the recast layer were then observed using an optical microscope (Keyence, VHX-6000, Osaka, Japan). This optical inspection provided an overall view of the recast layer’s morphology.

For a more in-depth microstructural analysis, the samples were etched with a nickel -based superalloy etchant (composed of 10% CuCl_2_ + 40% HCl + 50% ethanol). The etching process lasted for approximately 40 s until the sample surface turned gray. This color change indicated that the etching had reached an appropriate depth to reveal the microstructural features related to the recast layer. After etching, the samples were cleaned and dried using an ultrasonic cleaner (FUYANG, F-030ST, Hangzhou, China) to remove any residual etchant and contaminants.

Some samples were further processed for electron microscopy analysis. These samples underwent both mechanical and vibration-polishing to achieve a surface finish of 0.03–0.05 μm. This high-precision polishing was necessary to meet the requirements for detailed electron microscopy examination. Scanning electron microscopy (SEM) and energy-dispersive spectroscopy (EDS) were then performed using a lanthanum hexaboride SEM (TESCAN, Brno, Czechia, VEGA3) and a Raman-SEM (TESCAN, RISE-MAGNA). These techniques allowed for a more detailed analysis of the recast layer’s microstructure and elemental composition.

For the observation of fatigue fracture surfaces, both optical microscopy and SEM/EDS were directly employed. Optical microscopy provided a general overview of the fracture morphology, while SEM/EDS offered high-resolution imaging and elemental analysis, which were essential for understanding the failure mechanisms associated with the recast layer.

## 3. Results and Discussion

### 3.1. Microstructure and Mechanical Properties of the Recast Layer

#### 3.1.1. Microstructural and Compositional Analysis of the Recast Layer

**Figure 4:**
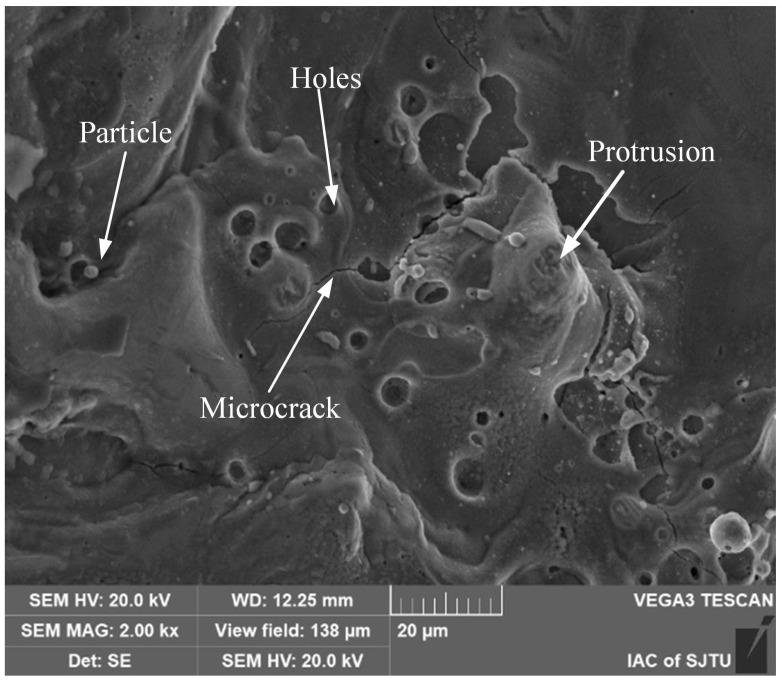
Electronic image of the inner wall of a small hole.

The recast layer is formed when the surface molten material cools and solidifies rapidly during the high-speed EDM process. It typically exhibits a flow-like raised surface, granular material, pores, and microcracks [22]. As shown in Figure 4, the electronic image of the inner wall of the micro-hole reveals these characteristics. The pores on the machined surface are formed due to the vaporization of the dielectric and the generation of metal vapor under high discharge temperatures. These gases become trapped within the molten metal. As the melt pool cools and solidifies rapidly, the trapped gases are unable to escape completely, resulting in the formation of pores. Cracks in the recast layer mainly occur because the discharge energy is transferred as heat to different depths of the machined surface. This leads to non-uniform temperature distributions across the workpiece. The thermal stress generated by this temperature gradient becomes residual stress after cooling. Notably, the recast layer and its adjacent areas experience high temperature gradients. As a result, significant tensile stress is induced, which can cause edge cracking. When this tensile stress exceeds the material’s tensile strength, cracks start to form.

**Figure 5:**
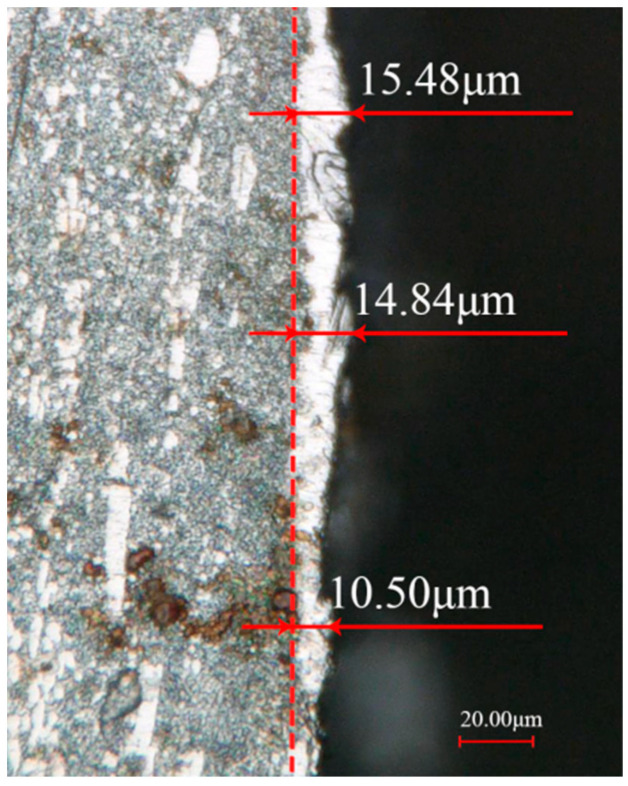
Thickness map of the recast layer on the inner surface of small holes machined by EDM with a pulse width of 50 μs.

Figure 5 shows the distribution of the recast layer on the inner wall of the hole. At a pulse width of 50 μs, the recast layer has an average thickness of approximately 14 μm. During the molten state and rapid solidification, the melt pool is influenced by the electrode and the surrounding environment. This influence causes changes in the composition and microstructure of the recast layer.

**Figure 6:**
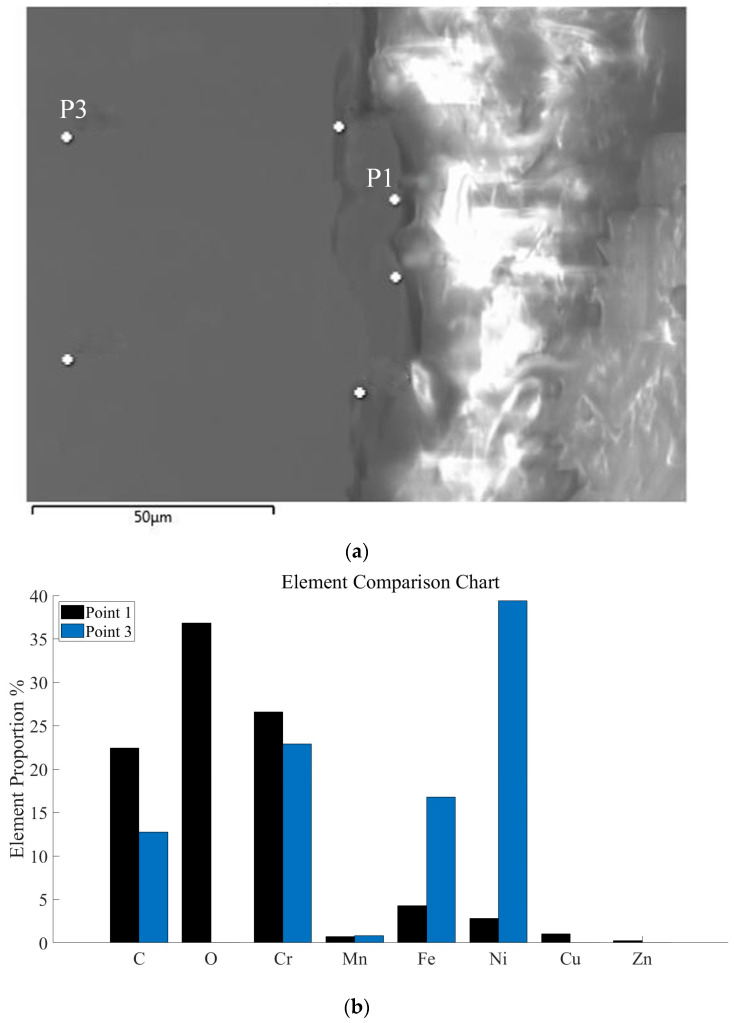
(**a**) EDS scanning of the recast layer and the substrate diagram and (**b**) distributions of major elements in the EDS surface sweep of the recast layer (P1) and base material (P3).

Figure 6 presents the elemental analysis results. Point 1, which is located in the recast layer, shows the presence of O, Zn, and Cu elements. In contrast, these elements are absent in Point 3, which is taken from the base material. Specifically, From Figure 6b, the content of O increased by 36.84% and that of Cu increased by 1.04% in the recast layer. Meanwhile, the content of Fe decreased by 12.54% and Ni decreased by 36.6%. In general, elements such as O, C, Cu, Cr, and Zn are enriched in the recast layer, while Ni and Fe are depleted. The recast layer is formed through the melting of surface material, creating a melt pool. In this melt pool, the base material and external elements can rapidly diffuse and mix. Cu from the electrode and O from the working fluid are present in higher concentrations in the recast layer. At high temperatures, Ni and Fe tend to oxidize and volatilize, leading to their reduced content in the recast layer.

#### 3.1.2. Mechanical Properties of the Recast Layer

**Figure 7:**
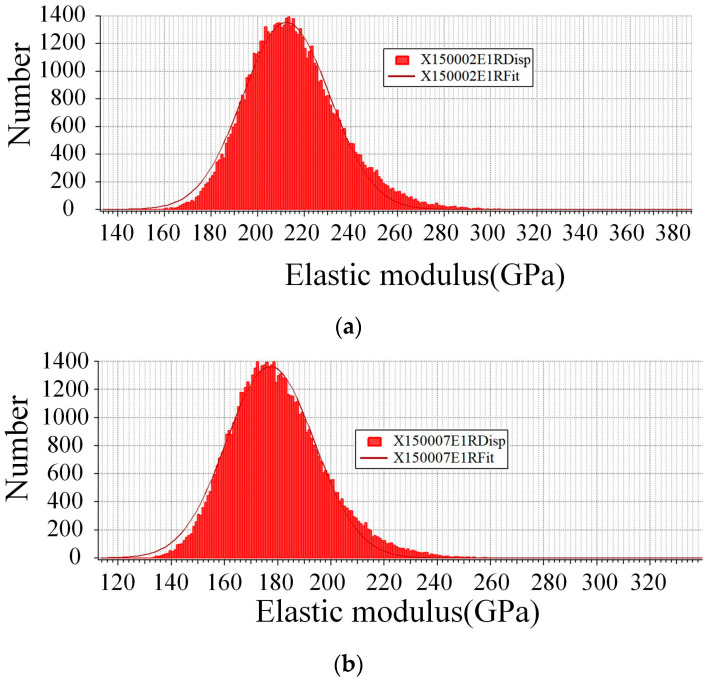
Modulus of elasticity of matrix (**a**) and recast layer (**b**).

Changes in the composition of the recast layer significantly modify its physical properties, particularly hardness and elastic modulus, both of which play crucial roles in determining the fatigue life of the material. Atomic force microscopy (AFM, MFP-3D) measurements [23], as depicted in Figure 7, indicate that the elastic modulus of the recast layer is 177 GPa, notably lower than that of the base material, which is 212 GPa. This reduction in elastic modulus can be attributed to the defects of microcracks and pores caused by rapid melting and solidification during the formation of the recast layer. The observed downward trend in elastic modulus is consistent with the test results presented by Newton et al. [24], yet it diverges from those reported by Roy et al. [25]. Furthermore, nanoindentation tests conducted using a nano-mechanical testing system (Hysitron TI-950) reveal that the average hardness of the recast layer is 4.77 GPa, compared to 3.96 GPa for the base material. This increase in hardness is mainly due to grain refinement and the formation of high-hardness compounds such as chromium oxide within the recast layer. This hardness trend aligns well with the findings detailed in the article [26], but it contradicts the results of another study [24]. Therefore, for different substrate materials or the same material processed under varying conditions, the physical properties of the recast layer exhibit significant differences. These differences underscore the complexity of the relationship between processing conditions, recast layer composition, and resulting physical properties.

### 3.2. Fatigue Fracture Analysis

**Figure 8:**
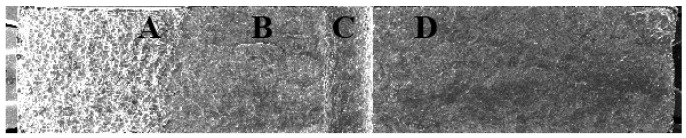
The fatigue fracture surface morphology of small holes processed by EDM magnified 28×.

The overall morphology of the ultrasonic fatigue fracture surface of microholes fabricated by EDM is presented in Figure 8. In this figure, C denotes the machined concave surface of the microhole. The regions labeled B and D represent the fatigue fracture surfaces that originated from ultrasonic fatigue, while A corresponds to the brittle fracture surface resulting from the impact testing.

Experiments reveals that defects within the recast layer induce material inhomogeneity [27] and local stress concentrations [28], ultimately resulting in fatigue failure. Fracture experiments demonstrate that, during the ultra-high-cycle fatigue of EDM-processed microholes, crack initiation sites are diverse. These sites include pre-existing microcracks, pores, inclusions present in the recast layer, and the interface between the recast layer and the base material.

#### 3.2.1. Initial Microcracks in the Recast Layer

**Figure 9:**
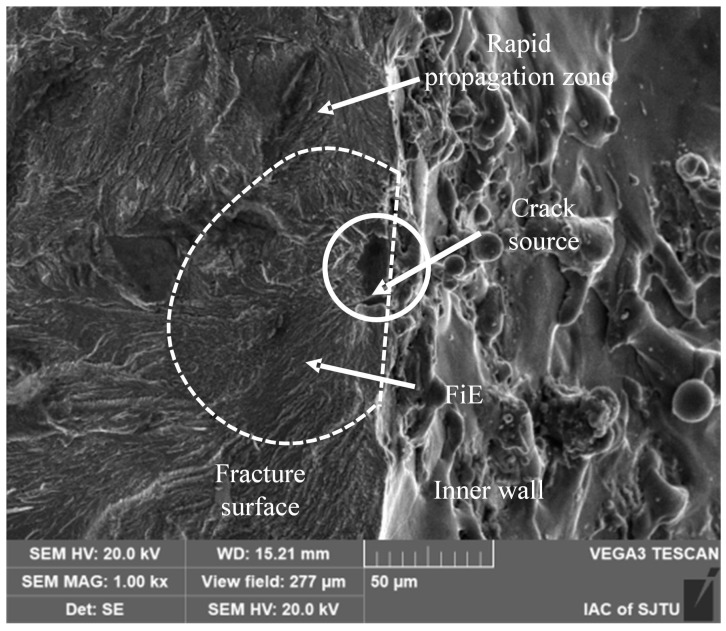
Initial microcracking crack in the recast layer.

As depicted in Figure 9, these pre-existing initial cracks act as the nucleation sites for fatigue cracks. Elemental distribution analysis in the vicinity of the crack initiation site reveals an enrichment of O and Cu elements. The crack entrance exhibits dimensions of approximately 2 μm in width and 20 μm in depth. Above the crack initiation site, a carbon-rich region is observed. This region was formed during the EDM process and is accompanied by an array of fine crack ridges. The presence of this carbonized region, along with its surrounding ridges and microcracks, gives rise to a rough crack initiation zone.

In the figure, the dashed line demarcates a relatively flat fatigue crack fisheye (FiE) region [29]. This region has a diameter of approximately 100 μm. Under electron microscopy, it appears darker and is characterized by uniform radial tear lines that emanate from the crack initiation zone. Beyond this fisheye region, the fracture surface features a rough fatigue crack stable propagation zone. This zone is formed as crack lines converge and transform into radial ridges, which are indicative of the progressive growth of the fatigue crack.

#### 3.2.2. Pore Defects in the Recast Layer

**Figure 10:**
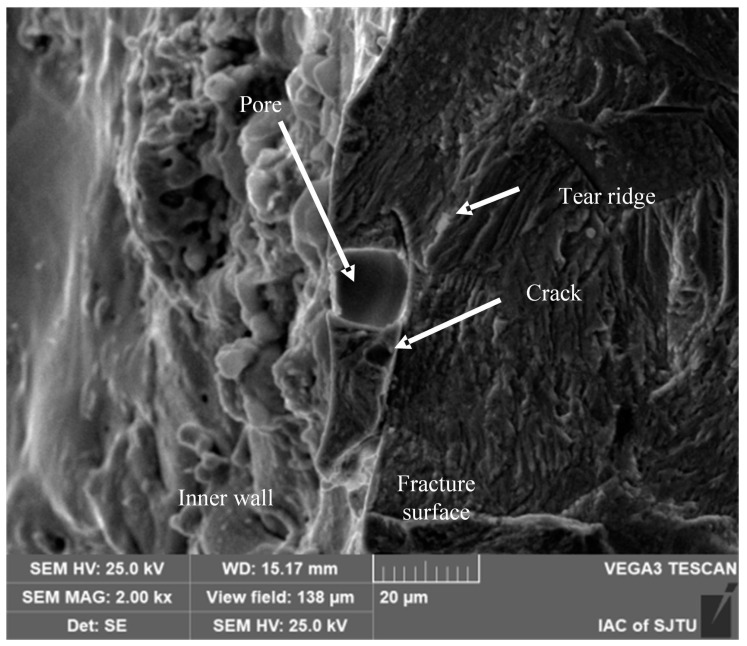
Initial hole crack source in recast layer.

Figure 10 presents the morphology of crack sources induced by pore defects in the recast layer at varying magnifications. The pores, with a diameter of approximately 15 μm, are spherical in shape and possess smooth surfaces. These pores were formed as a result of gas escaping from the molten material during the EDM process [25]. Elemental analysis of these pores reveals an enrichment of copper and oxygen. This compositional characteristic confirms that these pores are located within the recast layer rather than in the base material.

As shown in Figure 10, cracks and radial ridges are observed adjacent to the initial pore defects. Notably, the cracks propagate directly into the stable propagation zone without exhibiting the characteristic features of the fatigue crack FiE region. This observation indicates a distinct crack propagation mechanism associated with pore-induced crack initiation in the recast layer, which is different from the scenario where the FiE region is present.

#### 3.2.3. Inclusions in the Recast Layer

**Figure 11:**
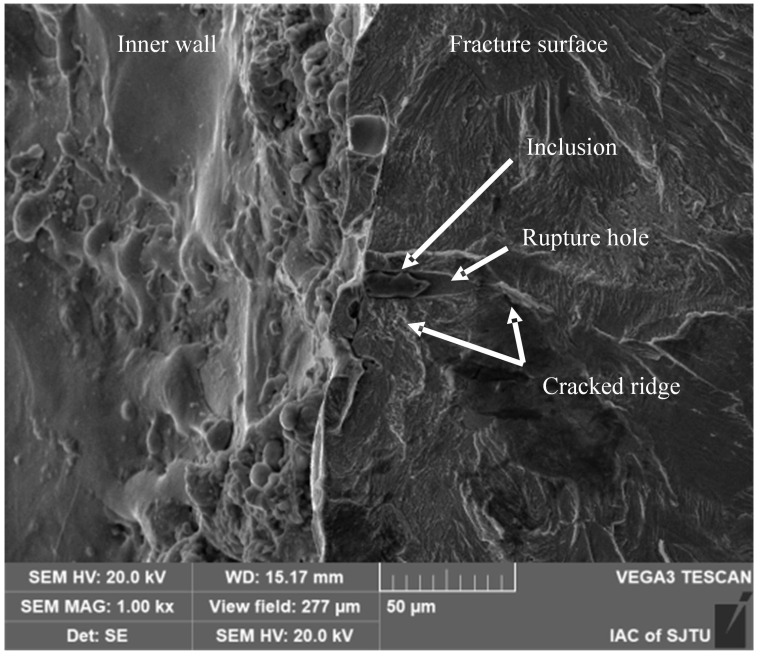
Recast layer inclusions crack source magnified 1000×.

**Figure 12:**
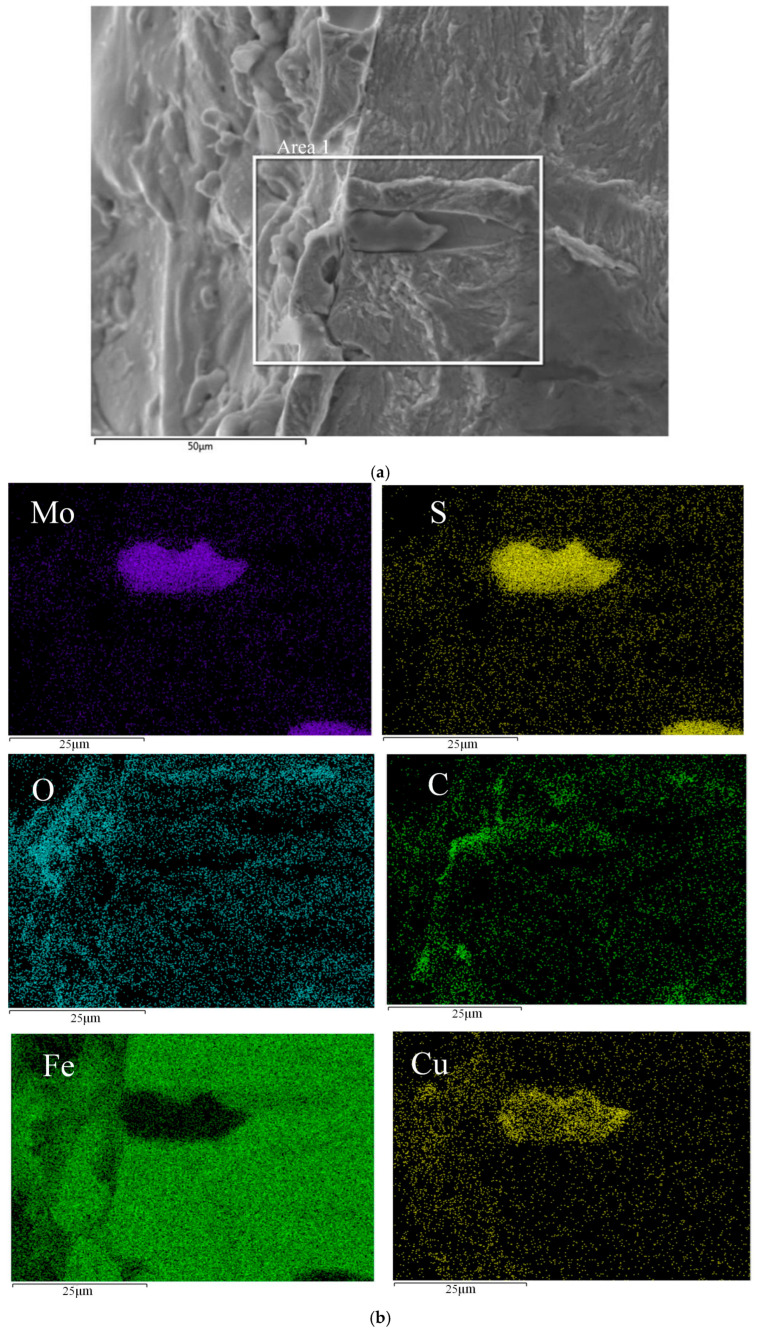
(**a**) Recast layer inclusions crack area and (**b**) elemental surface distribution of initial holes in the recast layer.

As depicted in Figure 11, elliptical tear holes are observed surrounding the inclusion. Elemental analysis, presented in Figure 12, reveals an enrichment of O and Cu elements on the left-hand side of the inclusion. This compositional feature confirms its location within the recast layer. The inclusion measures approximately 25 μm in length and 10 μm in width. High concentrations of Mo, S, and Cu are detected within the inclusion. Based on these elemental compositions, it can be inferred that the inclusion is a MoS precipitate phase within the GH3536 material. The presence of Cu in the inclusion is attributed to its introduction from the electrode during the high-temperature EDM process.

The elliptical tear holes are formed due to the local plastic deformation of the surrounding material during fatigue loading. Around the inclusion and tear holes, numerous radial ridges are present. The relatively large volume of the defect caused by the inclusion promotes direct crack propagation into the crack propagation zone, bypassing the formation of the characteristic fatigue crack fisheye (FiE) zone. This phenomenon indicates a unique crack-initiation and propagation mechanism associated with inclusions in the recast layer.

#### 3.2.4. Crack Initiation at the Recast Layer-Base Material Interface

**Figure 13:**
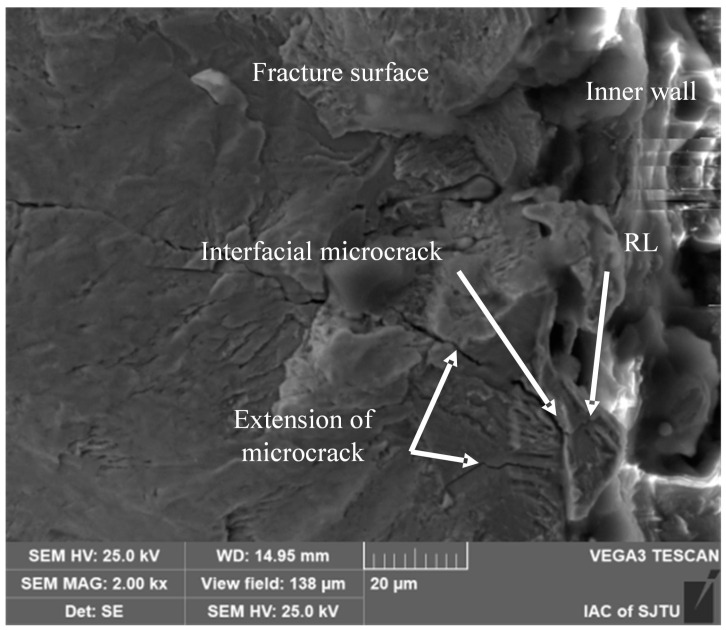
Source of cracks at the junction of recast layer and matrix.

Figure 13 illustrates the microscopic morphology of crack at the interface between the recast layer and the base material. Elemental analysis indicates an enrichment of O and Cu elements at the elevated region, which serves as a clear indication of the recast layer. The maximum thickness of this recast layer in this area is measured to be approximately 10 μm. Evidently, Figure 13 reveals a distinct crack precisely at the interface between the recast layer and the base material.

The formation of this crack can be ascribed to multiple contributing factors. Firstly, after EDM, due to the high discharge density, tensile residual stresses are generated on the surface [30]. These tensile residual stresses can lead to uneven stress distribution within the material, especially in areas with defects, interfaces, or other discontinuities, which are prone to becoming stress concentration hotspots. This stress concentration renders the interface highly vulnerable to crack initiation. Secondly, ultrasonic fatigue testing is predicated on specimen resonance. As detailed in Section 3.1, measurements have disclosed notable differences in elastic modulus and hardness between the recast layer and the base material. These property variances result in a mismatch of resonant frequencies between the two regions during fatigue testing. This frequency discrepancy, in turn, triggers interfacial debonding and ultimately gives rise to crack formation at the interface.

#### 3.2.5. Crack Formation in Recast Layers Without Apparent Defects

**Figure 14:**
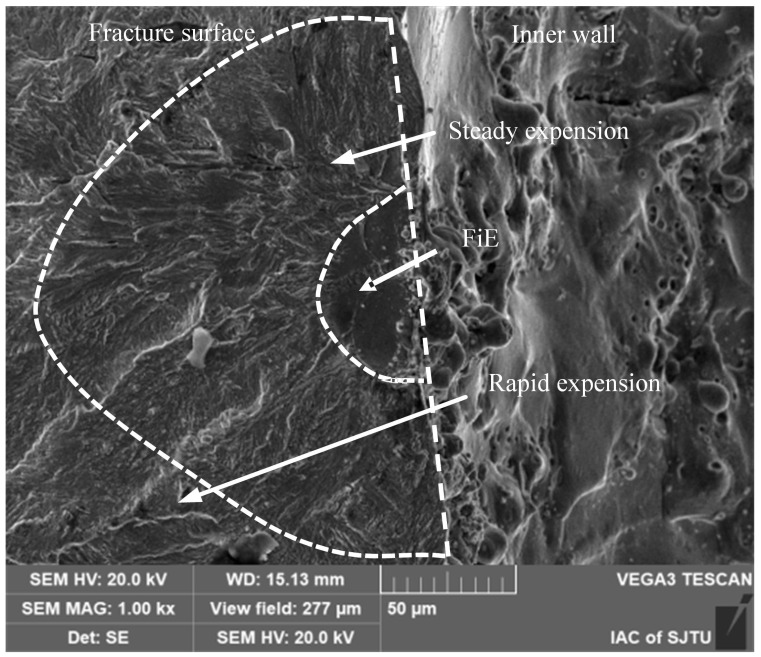
Source of defect-free cracks.

Figure 14 depicts the microscopic morphology of a crack that originated within the recast layer where no apparent structural defects are present. Elemental distribution analysis on the right-hand side of the crack source reveals an enrichment of O and Cu elements. This compositional feature firmly confirms the location of this region within the recast layer. Notably, in this particular area of the recast layer, there are no discernible structural defects such as pores or microcracks.

The fracture surface demonstrates distinct characteristics of a fisheye zone and a stable propagation stage. Under electron microscopy, the fisheye zone appears darker and is characterized by uniform radial tear lines that emanate from the crack initiation zone. The stable propagation zone is replete with numerous radial ridges that are formed as tear lines converge.

During the processing, the recast layer, which is formed by the random solidification of molten material, may not exhibit obvious structural defects. However, its non-uniform thickness leads to an increase in surface roughness. Local stress concentration occurs at surface depressions, rendering these areas highly susceptible to crack initiation under fatigue loading.

The ultrasonic fatigue fracture surfaces of high-speed EDM micro-holes consistently exhibit multi-source fracture characteristics. The crack sources are predominantly located in the vicinity of the micro-holes. This phenomenon can be attributed to the fact that the recast layer formed subsequent to micro-hole machining disrupts the stress distribution within the specimen. Specifically, it induces significant stress concentration around the hole. During the experimental process, processing defects within the recast layer propagate. As they do so, radial ridges are formed, with the crack source at the center. These ridges manifest as cleavage river patterns. The interaction of ridges stemming from multiple crack sources results in the formation of large tear ridges that are perpendicular to both the hole axis and the stress loading direction.

Notably, in contrast to traditional low-cycle and high-cycle fatigues, the fracture surfaces of very-high-cycle fatigue are relatively flat and oriented perpendicular to the stress loading direction. They lack the characteristic 45° macroscopic plastic slip fracture surfaces, as reported in [31].

**Figure 15:**
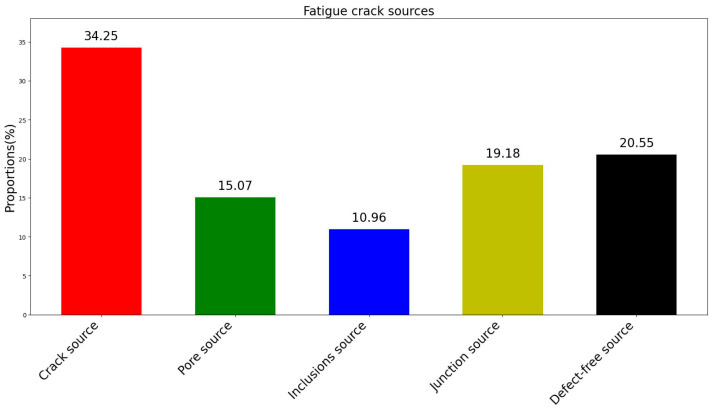
The proportion of ultra-high cycle fatigue crack sources.

The statistical analysis of crack sources is presented in Figure 15. Among the factors contributing to very-high-cycle fatigue, initial microcracks within the recast layer constitute 34.25%, making them the most probable precursors for the development of fatigue cracks. Initial pores, inclusions, and the interface between the recast layer and the base material account for 15.07%, 10.96%, and 19.18% respectively.

### 3.3. Very-High-Cycle Fatigue Life

To describe the influence of recast layer characteristics on ultrasonic fatigue failure, this section statistically analyzes the fatigue life of micro-hole specimens with recast layers, based on fatigue experiments. A total of 16 micro-hole specimens with recast layers were included in the analysis.

**Figure 16:**
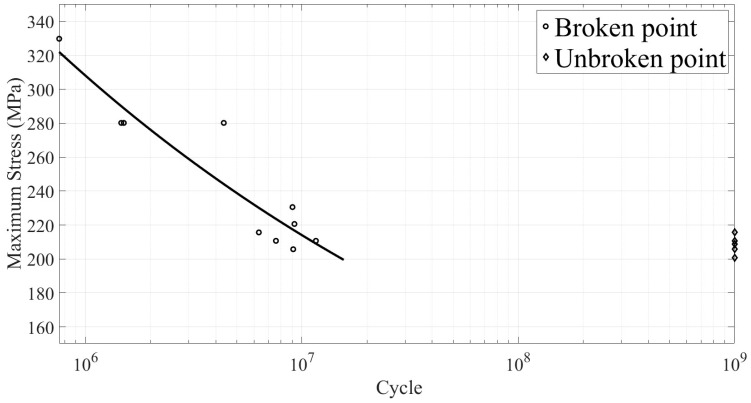
S–N Curve for high-temperature alloy GH3536 specimens with one small hole with a recast layer.

Figure 16 shows the stress–life (S–N) curve. Black circles represent the ultrasonic fatigue test results of recast layer (RL) specimens that failed, and black diamonds represent RL specimens that did not fail after 10^9^ cycles (unbroken).

Ultrasonic fatigue experiments conducted on microporous specimens with recast layers revealed two distinct characteristics. Specimens that failed exhibited fatigue lives concentrated below 5 × 10^7^ cycles, with the black life prediction curve fitted using the Basquin Equation (1). At Nf ≈ 10^7^ cycles under the same loading stress, some specimens did not fail after 10^9^ cycles, suggesting the existence of a fatigue limit.

(1) $$\sigma =2731.868\cdot {N_{f}}^{-0.158}$$

As explained in Section 3.2, fatigue cracks tend to start where stress is highest—specifically, at the thinnest part of the hole wall, perpendicular to the maximum stress. In theory, this is where cracks should form, leading to fatigue failure. However, the process of EDM introduces randomness. Each pulse of the EDM process creates a recast layer of varying thickness, with tiny cracks, holes, and impurities scattered unpredictably along the inner surface of the hole. If these recast layer defects happen to be in the high-stress zone, the specimen is more likely to fail early, as shown by the RL data points in the figure. On the other hand, if the high-stress area is free of major defects, the specimen can endure much longer, represented by the unbroken data points. By examining the fracture surfaces of both short-life and long-life specimens, it is clear that short-life failures are caused by defects in the recast layer. In contrast, long-life failures often have crack origins that are not linked to the recast layer at all.

### 3.4. Fatigue Failure Mechanism of High-Speed EDM Recast Layer Micro-Holes

Fracture analysis of very-high-cycle fatigue experiments reveals that crack initiation is not induced by alterations in the physical properties of the recast layer. Instead, it is primarily attributed to surface defects on the inner wall of the hole, such as pre-existing cracks, pores, and inclusions, along with debonding at the interface between the recast layer and the base material. The fatigue behavior at the recast layer-base material interface is significantly influenced by variations in resonant frequency. These frequency changes stem from disparities in elastic modulus and residual stress introduced during the machining process.

Under cyclic loading, stress concentration at the hole edge plays a crucial role in determining the macroscopic location of fatigue initiation. Typically, fatigue initiates in the vicinity of the maximum hole diameter, perpendicular to the loading direction. Nevertheless, the specific distribution of fatigue sources is intricately governed by the competitive interplay between structural defects (e.g., initial cracks, pores, and inclusions) within the recast layer and the recast layer-base material interface.

## 4. Conclusions

This study investigated the ultrasonic fatigue behavior and fracture mechanisms of small-hole specimens, manufactured using high-speed EDM. The primary focus was to analyze fatigue crack initiation and fracture characteristics through the examination of fracture morphology. The key findings of this study are as follows:

(1) Recast Layer and Base Material Differences: The recast layer produced by high-speed EDM differs significantly from the base material in terms of elemental composition, elastic modulus, and hardness. Specifically, the recast layer exhibits higher hardness (4.77 GPa) compared to the base material (3.96 GPa), reflecting a 20.4% increase. However, the elastic modulus of the recast layer (177 GPa) is lower than that of the base material (212 GPa), representing a decrease of approximately 16.5%.
(2) Fracture Morphology: The fracture observations reveal that all small-hole specimens exhibited multi-source fracture characteristics during ultrasonic fatigue testing. Fatigue cracks were found to initiate at various axial positions along the air-film cooling holes.
(3) Fatigue Crack Initiation in Recast Layer Specimens: In specimens with a recast layer, fatigue crack initiation predominantly occurred in five regions. The first category involves areas with initial defects, where crack initiation sites included initial microcracks within the recast layer (34.25%), pores within the recast layer (15.07%), and inclusions within the recast layer (10.96%). The second category involves regions without clear initial defects, where cracks primarily initiated at the interface between the recast layer and the base material (19.18%) or in areas without obvious structural defects (20.54%). These initiation regions competed with one another, ultimately determining the site of initial fatigue crack formation.
(4) Fatigue Crack Initiation Without Obvious Initial Defects: Even in the absence of noticeable initial defects, crack initiation was significantly influenced by the characteristics of the recast layer. The differences in composition and microstructure between the recast layer and base material result in significant variations in elastic modulus and hardness. This makes the interface between the two materials prone to high residual stresses, behaving similarly to a joint between dissimilar materials. Furthermore, the resonance frequency mismatch between materials with differing elastic moduli may exacerbate crack initiation at the interface. Additionally, the recast layer’s increased surface roughness promotes the initiation of fatigue cracks.
(5) The recast layer (RL) generated during EDM significantly impacts the fatigue performance of materials. Randomly distributed defects within the RL are the primary cause of variations in fatigue life. When defects in the RL are present in high-stress regions, specimens are prone to early fatigue failure, often occurring before 5 × 10^7^ cycles. Conversely, when high-stress regions are free of noticeable defects, specimens can endure higher cycle counts, potentially reaching up to 10^9^ cycles without failure, suggesting the existence of a fatigue limit. Therefore, the quality of the recast layer and its distribution in high-stress areas are critical factors determining fatigue life.

## Figures and Tables

**Table 1:** Chemical composition of GH3536.

Element	C	Cr	Ni	Co	W	Mo	Fe
Content/wt.%	0.05–0.15	20.50–23.00	other	0.50–2.50	0.20–1.00	8.00–10.00	17.00–20.00

**Table 2:** GH3536 Mechanical properties.

Elastic Modulus E (Gpa)	Density ρ(kg/m^3^)	Poisson’s Ratio μ	Tensile Strength σ_b_ (MPa)	Yield Strength σ_s_ (MPa)
206	8.28 × 10^3^	0.3	690	275

**Table 3:** Basic parameters for high-speed EDM of small holes.

Type	*D* (mm)	T_on_ (μs)	T_off_ (μs)	I_A_ (A)	C (μF)	*r* (min)
RL	0.57	50	10	3	0.172	200

## Data Availability

The raw data supporting the conclusions of this article will be made available by the authors on request.
